# External Human–Machine Interfaces for Automated Vehicles in Shared Spaces: A Review of the Human–Computer Interaction Literature

**DOI:** 10.3390/s23094454

**Published:** 2023-05-02

**Authors:** Sarah Brill, William Payre, Ashim Debnath, Ben Horan, Stewart Birrell

**Affiliations:** 1 Centre for Future Transport and Cities, Coventry University, Coventry CV1 5FB, UK; 2 Faculty of Science, Engineering and Built Environment, Deakin University, Waurn Ponds, VIC 3216, Australia

**Keywords:** automated vehicle, external human–machine interface, shared space vulnerable road user

## Abstract

Given the rise of automated vehicles from an engineering and technical perspective, there has been increased research interest concerning the Human and Computer Interactions (HCI) between vulnerable road users (VRUs, such as cyclists and pedestrians) and automated vehicles. As with all HCI challenges, clear communication and a common understanding—in this application of shared road usage—is critical in order to reduce conflicts and crashes between the VRUs and automated vehicles. In an effort to solve this communication challenge, various external human–machine interface (eHMI) solutions have been developed and tested across the world. This paper presents a timely critical review of the literature on the communication between automated vehicles and VRUs in shared spaces. Recent developments will be explored and studies analyzing their effectiveness will be presented, including the innovative use of Virtual Reality (VR) for user assessments. This paper provides insight into several gaps in the eHMI literature and directions for future research, including the need to further research eHMI effects on cyclists, investigate the negative effects of eHMIs, and address the technical challenges of eHMI implementation. Furthermore, it has been underlined that there is a lack of research into the use of eHMIs in shared spaces, where the communication and interaction needs differ from conventional roads.

## 1. Introduction

Vulnerable road users (VRUs)—such as pedestrians and cyclists—account for more than half of the road fatalities as they are unprotected by an external shield (e.g., a vehicle body) in traffic [1]. Despite the lack of empirical evidence, automated vehicles (AVs) are often touted as a solution to improve the safety of other road users through the reduction of human error in road use. Given the rise of AVs from an engineering and technical perspective [2], there has been increased research interest concerning the Human and Computer Interactions (HCI) between VRUs and AVs.

From a Human Factors and HCI perspective, making the decisions of AVs clear to all road users will enable their smooth integration into traffic [3]. Therefore, a solution that has been explored in scientific publications, media, and industry concepts is the use of external human–machine interfaces (eHMIs) [4]. The eHMIs are advanced communication interfaces that inform pedestrians of the current state and future actions of a vehicle [5]. They have shown promise in resolving ambiguity, maximizing efficiency, and increasing pedestrians’ feelings of trust and safety in AVs, particularly in negotiation situations between AVs and pedestrians [6]. Additionally, eHMIs may reduce the accident risk that results from failures in communication [7]. 

With the abundant number of studies investigating eHMIs-road users’ interactions, there is a need to understand what has been covered by the scientific literature and what has yet to be explored. Furthermore, it is important to understand how communication between AVs and VRUs in such interactions can be made straightforward and less ambiguous in shared spaces—a type of road space where such interactions are critical from a safety point of view. As a result, the research questions of the present study are as follows: *What do we know about eHMIs-road users’ interactions, particularly in the context of shared spaces? What are the gaps in the human–computer interaction literature with respect to this topic?*

### 1.1. Related Work

In a recent review, Ref. [8] aimed to advance the discussion of whether eHMIs are necessary, which presented arguments covering both the positive and negative effects of eHMI inclusion. While eHMIs are generally desired by VRUs and have been shown to support safe crossing decisions, Ref. [8] noted that with repeated exposure VRUs may begin to over-rely on the device which could result in dangerous situations. Across academia and industry, several eHMI concepts have been proposed. With the aim of resolving the lack of standardization between them, Ref. [4] developed an 18-parameter taxonomy based on 70 eHMI concepts to provide a structure for researchers to use when describing future concepts according to their physical and functional characteristics. Additionally, Ref. [3] further explored the regulations governing eHMIs, the frequently occurring characteristics of the different eHMI concepts that exist within the literature, and the evaluation methodologies. Finally, Ref. [9] detailed the current knowledge surrounding pedestrian behaviors in shared spaces. Each of these reviews contributes to the understanding of the benefits of eHMIs, and the current state of eHMI design and development. Despite their contributions, the existing reviews exhibit limitations. In particular, these reviews provided limited focus and details on the research into AVs in shared spaces, specifically how communication between AVs and VRUs in shared spaces could be made straightforward and less ambiguous for improving the safety of VRUs. This paper covers several key themes, including the following: (1) AV-VRU communication; (2) the design and evaluation of eHMIs; (3) the use of eHMIs in shared spaces.

### 1.2. Objectives

This paper presents a critical review of the interaction between vulnerable road users (VRUs) and automated vehicles (AVs) from an HCI perspective in the context of shared spaces, bridging the gap between human factors and emerging technologies literature. This paper identifies topics that have yet to be explored and areas for future research. 

The contribution of the present review is threefold, as outlined below:

1. It provides an overview of the current and fast-growing research into eHMI design and evaluation focusing on how vehicles and VRUs communicate, the characteristics of external human–machine interface technologies, and the subsequent evaluation of the interfaces.
2. In addition to the research overview, it provides a critical review of the applications of various VRU-vehicle communication and eHMI technologies in shared spaces.
3. More importantly, it highlights research gaps within the literature surrounding the use of eHMIs for VRU-AV communication and interaction and provides recommendations for the direction of future research.

### 1.3. Vulnerable Road Users

Active transport modes, such as walking or cycling, provide health and environmental benefits [10,11]. Additionally, due to the COVID pandemic, a decrease in the use of public transport, and increased use of active transport modes and private cars were observed due to the fear of infection [12,13,14]. Active transport allowed people to socially distance themselves while having the added benefit of maintaining their physical and mental wellbeing through an increase in physical activity.

While this increase in active transport is generally viewed positively, the safety of VRUs is an area of concern [15,16]. Vulnerable road user is a term that is applied to those who are unprotected by an external shield in traffic, making them three to four times more prone to injury in any vehicular collision [11,17,18]. This includes pedestrians, bicyclists, motorcyclists, and kick-scooter users. According to the latest global assessment of road safety, the World Health Organization (WHO) indicates that VRUs account for more than half of all road fatalities [1]. Specifically, in 2020 in the UK and Australia, pedestrians and cyclists accounted for approximately 33% and 16% of all road deaths, respectively [19,20]. In both Australia and the UK, cyclists were the only vulnerable road user group to see an increase in fatalities from pre-pandemic levels. This may be due to the increase in cycling as a mode of transport due to the range of health and leisure benefits in addition to the COVID pandemic [20,21]. As active transport continues to increase, traffic fatalities of VRUs are a growing public health concern that must be addressed.

### 1.4. Automated Vehicles

In 2015, the United States Department of Transportation’s National Highway Traffic Safety Administration (NHTSA) published a report on critical reasons for crashes nationwide [22]. In 94% of the events, the critical reason for the crash was assigned to the driver and 85% attributed to human error. Several pieces of published research have cited the 94% statistic from the NHTSA report to suggest that AVs have the potential to minimize traffic fatalities through reductions in human error [18,23,24,25,26]. The improvements that AVs could bring to road safety include the mitigation of lapses in drivers’ attention, improvements in response times, and the inclusion of pedestrian protection systems, which reduce harm by deploying active braking or pedestrian airbags when an unavoidable collision is detected [23,27,28]. Similarly to manual vehicles, automated vehicles (AVs) can be separated into different vehicle categories. They can range from passenger vehicles that resemble manual vehicles to ‘minicars’ (often called “pods” in the literature). These pods are low-speed automated vehicles (LSAVS) that rarely exceed 20 mph and do not mix with high-speed traffic [29].

However, the interactions between the AV and surrounding traffic participants could create more complexities in the traffic system. In future automated vehicles, the driver’s seat may be empty, and the ‘driver’ may not be visible or may be engaged in other activities. As AVs are introduced into traffic, the lack of established conventional communication, such as eye contact or hand signs, is likely to lead to frustrations for VRUs. Currently, traffic interactions stem from expectations and experiences based on existing traffic rules, the design of the road, and the current behavior of the road user [11,30]. As such, communication between road users plays a significant role in safety. Clearly informing VRUs of a vehicle’s intentions is an invaluable component for improving road safety as research has found that misinterpretation, or “faulty diagnoses”, of traffic situations by VRUs contributes to collisions [31]. Ref. [32] found that approximately 53% of pedestrian and 35% of cyclist collisions with vehicles that were analyzed were caused by a lack of critical safety information being signaled to, or recognized by, the VRU. The use of indicator lights, the road layout, and the size, speed, and distance of the vehicle, are all used by pedestrians to predict the drivers’ intentions and make road crossing decisions [33,34]. As such, road safety is not only determined by the Avs’ ability to perform infallibly but is also determined by the outcome of the interaction between AVs and other road users. This is especially true in areas where there are not formal traffic rules, such as shared spaces [27,35]. This will not only create awkward interactions but will also increase the amount of ‘standstill’ interactions between VRUs and AVs [28,36]. 

### 1.5. Shared Spaces

The current development and introduction of automated pods will often take place in shared spaces, such as university campuses or city centers, and will often cover intermediate distances, or the ‘last mile’ of a journey [27,37]. As such, shared spaces have been highlighted as an important research area in the development of automated vehicles. Shared spaces are implemented for a number of reasons, including clean air zones and carbon reduction within cities, thus facilitating active transport, managing traffic congestion, and reducing the risk of vehicular injuries to pedestrians and cyclists [38]. While in the UK cyclist traffic increased by 46% in 2020 compared to 2019, researchers agree that in order to maintain this shift to active transport there must be a change in pedestrian and cyclist infrastructure [12,13,14,39,40]. Shared spaces have been suggested as a solution to maintain this change.

A common goal of many urban design theories is giving the streets back to pedestrians [41]. The UK Department for Transport [40] defines a shared space as follows:

*A street or place designed to improve pedestrian movement and comfort by reducing the dominance of motor vehicles and enabling all users to share the space rather than follow the clearly defined rules implied by more conventional designs (p. 6–currently suspended).*

A key hallmark of shared spaces is that the priority between users is not governed by conventional traffic devices or formal traffic rules; rather, they are governed by social interactions between road users.

#### 1.5.1. Types of Shared Spaces

Before discussing the interactions within shared spaces, it is essential to understand what shared spaces are, and the several forms they can take. In a shared space, curbs and centerlines are removed to give the impression of a plaza or public square, blurring the segregation between vehicle drivers and people walking, cycling, or scootering. Shared spaces also generally include features to humanize the space (i.e., benches, street trees, and light fixtures), and areas where people may sit and socialize [42]. All of the above features contribute to creating spatial ambiguity within the space to keep drivers alert and to feel more welcoming to the most vulnerable road users. However, not all shared spaces are created equally. As they are implemented for a variety of reasons, the features of the space may vary depending on the culture and context in which the scheme is implemented [43]. Different shared spaces may use different design principles, such as differing levels of demarcation between roads, footpaths, and restrictions of vehicle speeds through a speed limit or street design. According to the DfT, two major factors can be used to differentiate one shared space from another. The first is the level of separation between vehicles on the carriageway and vulnerable road users on the sidewalk. The second factor is the number of interactions that occur between users. The more interaction that occurs, the more shared the space is [40,43].

**Figure 1:**
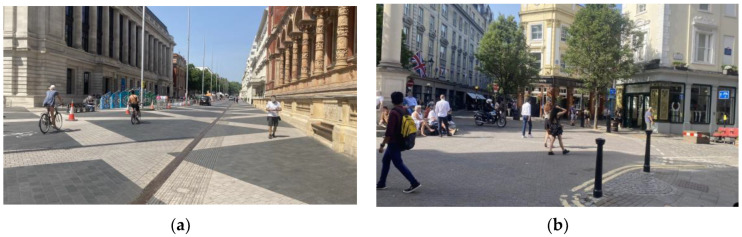
Examples of Pedestrian Priority Streets: (**a**) Exhibition Road, London, UK; (**b**) Seven Dials, London, UK.

**Figure 2:**
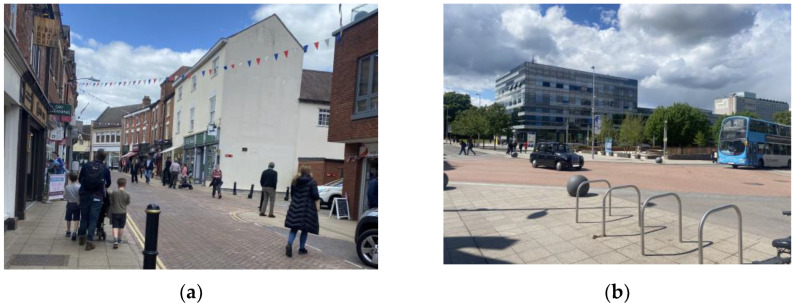
Examples of Informal Streets: (**a**) Swan Street, Warwick, UK; (**b**) Intersection of Gosford Street and Cox Street, Coventry, UK.

**Figure 3:**
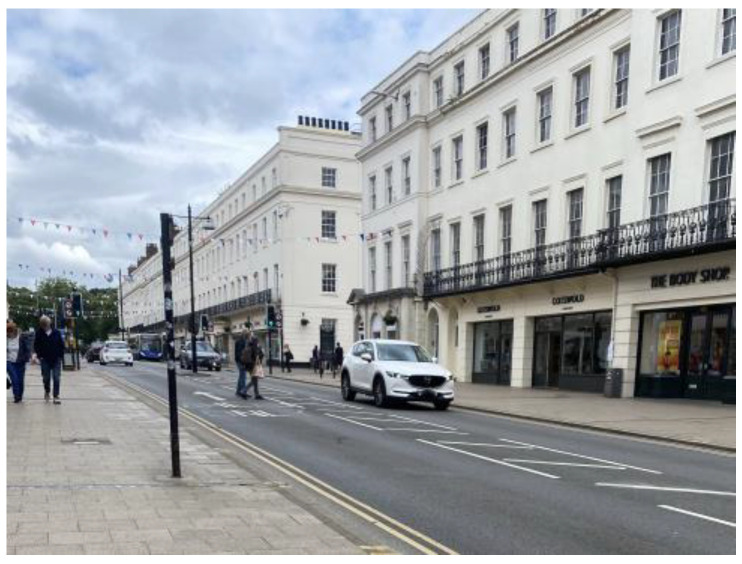
Example of an Enhanced Street. The Parade, Royal Leamington Spa, UK.

On the “least shared” end of the spectrum, pedestrians and vehicles are separated by curbs, painted lines, or pedestrian barriers; they will primarily interact at formal crosswalks. In the “most shared” places there is little to no demarcation between VRUs, and vehicles and road users may interact in any part of the space. The Charted Institution of Highways and Transportation identified three broad types of street design to classify the differences in shared spaces [44]. They are as follows:

- Pedestrian Prioritized Street (PPS): PPSs will not have well-defined carriageways so users of the space do not assume that pedestrians must cross at a defined crosswalk or seek drivers’ consent to cross. To emphasize the space as a place to be enjoyed, PPSs will generally have a level surface made up of similar paving types and colors across the whole of the space, and seating or other street furniture placed in the street. An example of a PPS can be seen in Figure 1.

- Informal Street: Informal streets will generally have a defined carriageway but will have an absence of or reduction in formal traffic control measures (such as traffic signals or zebra crossings), particularly at junctions [38,40]. While these spaces can in-principle contain dedicated cycling infrastructure, such as cycling lanes, they are not a hallmark of the space. An example of an informal street and an informal junction can be seen in Figure 2.

- Enhanced Street: Enhanced streets are on the limit of what can be called a shared space; they are conventional streets where care has been taken to improve the quality of the space through the removal of unnecessary street clutter and the introduction of features such as seating or street trees. Conventional traffic engineering features, such as junctions controlled by traffic signals or give way markings, are retained. An example of an enhanced street can be seen in Figure 3.

However, there is no agreed-upon definition of a shared space in the literature. A lack of understanding surrounding the dimensions of shared spaces has prevented a coherent definition of all of the different aspects of shared spaces [45]. A consistent taxonomy of shared spaces could help in evaluation, as behaviors in more shared spaces will not necessarily translate to the least shared spaces.

#### 1.5.2. VRU-Vehicle Interactions in Shared Spaces

Due to the lack of formal traffic rules or established behavioral norms, interactions within shared spaces may be ambiguous, where the intended actions of the road users involved are unclear [37]. Reference [46] compared shared spaces to an ice rink where users negotiate activities through “an intricate and unspoken set of protocols” (p. 169). As such, interactions within a shared space require more cooperation and negotiation between users than conventional roads do; users are required to work together to determine matters of priority and right of way. 

Through video analysis of Exhibition Road in London before and after its redevelopment into a shared space, Ref. [47] evaluated changes in pedestrian gap acceptance. Following the addition of shared space elements, pedestrians accepted a shorter gap in traffic and appeared more at ease when crossing. These results suggest that pedestrians feel more comfortable and confident in their interactions with vehicles in shared spaces when compared to conventional roads. Ref. [29] utilized virtual reality head-mounted displays to evaluate pedestrian gap acceptance in multiple environments (including a shared space) in response to a platoon of pods traveling at low speeds, followed by semi-structured interviews to help give subjective context to the gap acceptance results. Results from this study indicate that pedestrians are more willing to accept a gap of the same size in a shared space when compared to a single-lane road environment. However, as the speed of the vehicles increased, the gap acceptance converged for both environments. Additionally, interviews revealed that pedestrians reported lower safety scores in the shared space as they were unsure of the vehicle’s intended path. Similarly, using virtual reality, Ref. [48] evaluated pedestrians’ attitudes towards interacting with an AV in a shared space in 18 different scenarios. In summary, participants indicated increased feelings of safety when the pod was farther away and traveling more slowly. However, the distance had more of a significant effect than the pods’ speed. In video analyses of three different locations where automated vehicles were deployed, pedestrians avoided having to closely interact with AVs in a shared space, even changing their trajectory to move out of the AVs’ path [49]. However, pedestrians on conventional roads were likely to cross ahead of the AV, likely due to the presence of zebra crossings. 

Despite cyclists being identified as a key road user group affected by street design, much of the presently published work has focused on pedestrian behaviors. Using video observations, Ref. [50] analyzed how the redevelopment of Exhibition Road in London, UK, into a shared space has changed cyclists’ behavior, followed by a survey investigating cyclists’ perceptions of the new shared space. Results from the video observations and analysis indicated that the shared space has attracted more cyclists to the area while also reducing the average cycling speed. The survey results indicate that the added elements of a shared space, notably the pavement surface and the provision of bicycle facilities, increased perceived ease of movement and perceived safety. However, many cyclists expressed confusion about where they were able to cycle. Similarly, using video observations, Ref. [51] evaluated cyclists’ behavior in both a shared space and a non-shared space junction in Coventry, UK. The analysis uncovered that cyclists navigate both shared and non-shared junctions by cycling on the crosswalk or the sidewalk. Given that cyclist behavior is linked to their perception of roadway conditions, this may indicate that cyclists feel it is uncomfortable or inefficient to share the space with vehicles at junctions. Similarly, Ref. [52] found that cyclists experienced various challenges related to road infrastructure and were skeptical about sharing the road with automated vehicles, particularly in shared environments.

## 2. Methodology

To meet the first objective, an online keyword search was performed using AND/OR operators across the main concepts of the research topic, including “automated vehicle”, “autonomous vehicle”, “driverless vehicle”, “self-driving vehicle”, “automated car”, “autonomous car”, “driverless car”, “self-driving car”, “external human–machine interface”, “eHMI”, “external HMI”, “vulnerable road user”, “pedestrian”, “cyclist”, “bicyclist”, and “scooter.”

As of March 2023, a search of the aforementioned keywords returned 137 results within Scopus, a database that links to ScienceDirect, IEEE Xplore, and MDPI. As a result of the large number of publications available, this review of this literature is not exhaustive. Instead, the aim of this search was to complete a comprehensive review to identify gaps in the research.

To meet the second objective, a second search was performed to identify relevant studies researching the use of eHMIs in shared spaces. The keywords from the former search were utilized in addition to the following: “shared space”, “shared zone”, and “pedestrianized”.

**Figure 4:**
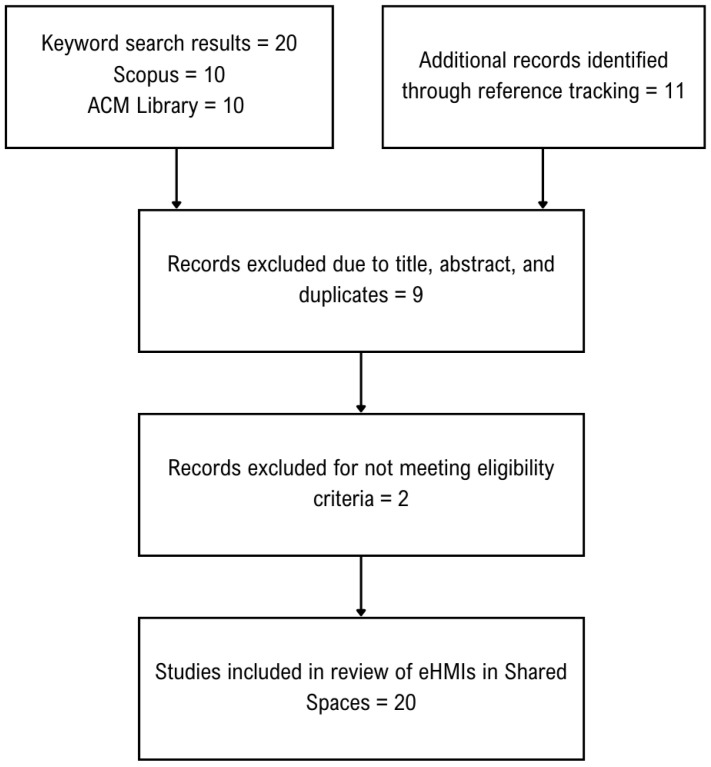
Flowchart of the selection process.

The selection process for this review was according to PRISMA guidelines and has been depicted in Figure 4 [53]. We accessed Scopus as the main database but also explored papers from the ACM library. Following a review of the papers, reference tracking was performed to identify additional studies. 

The screening was first performed on the title, abstracts, and keywords, and for those papers that appeared potentially relevant, the full papers were browsed to ascertain the fulfillment of the criteria. Papers were chosen based on the following criteria:

1. Papers in peer-reviewed journals, peer-reviewed conference proceedings, and reports of normal academic standards;
2. Written in the English Language;
3. The full text was accessible.

The search was focused on a human factors point of view; therefore, results from a technical frame of reference were excluded. A single screening procedure was adopted, where one person identified and selected papers from the relevant databases, and then assessed their eligibility according to the review criteria outlined. Whilst double screening has been suggested to reduce missed papers by around 5% [54], this could be considered more necessary for systematic reviews in a broad area of research interest. Single screening, with its benefits of time to complete and reviewer consistency, is considered an appropriate methodology, especially given the emerging nature of research in the field of eHMI use in shared spaces. Data were analyzed using a narrative synthesis approach to summarize the state of knowledge, identify gaps, and provide a direction for future knowledge.

## 3. Results

In this section we present the results of the literature research, beginning with a general review of VRU-Vehicle communication on both conventional roads, followed by a review of the general findings of eHMI use on conventional roads, and lastly a review of the use of eHMIs in shared spaces.

### 3.1. External Human—Machine Interfaces

VRUs employ a variety of different communication cues to resolve ambiguities during interactions which results in a challenge to determine the ideal communication method that AVs should adopt. Road users consider many different factors when deciding to cross a road, including vehicle speed, group size, eye contact, age, and the presence of formal traffic laws and control devices (i.e., crosswalk signals) [35,54]. While research surrounding the ideal communication method is complex, researchers generally agree that road users were more willing to cross if the driver acknowledges them in some way, through either implicit or explicit communication [37].

### 3.2. VRU-Vehicle Communication

Aside from the formal rules regulating the road, there are several informal rules governing road users’ interactions and communication with one another. According to [55], explicit communication methods, such as eye contact or hand gestures, are done intentionally by the sender to communicate a message to the receiver. Conversely, vehicle intention can be inferred through implicit communication methods, such as vehicle movement patterns, in which behaviors may be conducted without the intent of communication.

In both a simulated virtual environment and in the field, recent research has demonstrated that implicit communication can provide enough information for a pedestrian to decide whether to cross [35,56]. They propose that the vehicle motion itself can serve as a sufficient signal for pedestrians to cross as it mimics existing interaction patterns. In re-search conducted by [35], pedestrians felt equally safe interacting with AVs as with manual vehicles when only implicit information was available. In a field study, participants in [57] were more likely to base their crossing decisions on the distance between passing cars and the general environmental context than the use of an explicit eHMI display. Additionally, through onsite observations, Ref. [55] discovered that in non-automated vehicles, explicit communication is rarely used. As such, it is questioned whether eHMIs are necessary, and argued that implicit signals may be sufficient in communicating the vehicle’s intention [35,55,57]. 

However, research has also shown that communication via vehicle movement is not sufficient in all circumstances. As such, researchers have theorized that greater explicit communication may be required, specifically in more complex situations where priority is unclear [27,35]. Additionally, when the expected behaviors of a vehicle are not met, pedestrians seek to engage in explicit communication [55]. References [58,59,60] uncovered that people used explicit communication, particularly eye contact, to communicate with drivers.

Several studies posit that implicit and explicit communications are interdependent and that for optimal interaction, the message of the eHMI must match the implicit communication [6,37,56]. A participant in the Wizard-of-Oz study conducted by [61] noted that “it is not only about eye contact, it is the entire behavior of the driver that I usually look at” (p. 12). Wizard-of-Oz studies give the participants the impression that the vehicle is automated when it is actually controlled by a human operator. This can be achieved by using a “car seat costume” that conceals the human driver, by having a dummy steering wheel, or by putting artificial LiDAR sensors on the roof of the vehicle [28,62,63]. If the two communication methods do not align, the explicit communication provided may cause road users to ignore implicit cues and engage in risky behavior [56].

However, both implicit and explicit communication signals can be ambiguous and can lead to potential conflicts and a reduction in safety if they are misinterpreted [37]. For example, cultural differences may cause different interpretations of explicit communication; a honk of a horn may be considered an expression of irritation in the UK and the USA, whereas in China it is used in a friendly manner, and in Southern Europe, it may be used to alert other road users that a vehicle will accelerate [37]. Furthermore, it is argued that eHMIs may also have unintended consequences. A major safety concern is that road users may form inappropriate attitudes and behaviors towards automated vehicles [7]. For example, if road users purely rely on information from the eHMI, they may fail to actively evaluate their surroundings to assess whether it is safe to cross [7,35]. To further clarify this point, Ref. [64] found that “participants felt interfaces reduced their responsibility in making crossing decisions” (p. 9). Additionally, the addition of new signals will add to the road users’ cognitive load and may cause information overload; this may be exacerbated if the signals are non-intuitive and confuse the road users [35].

### 3.3. eHMI Technologies

The potential implementations of eHMIs are manifold; they range from visual, which is the most common, to auditory, which occurs rarely, to haptic, which has hardly been explored. The best modality, messaging, color, and position are all currently unclear [4,7,36]. As previously stated, eHMIs are considered a good method of explicit communication when no driver, or one engaged in non-driving related tasks, is present in an auto-mated vehicle. Across both academic and industrial research, many eHMIs concepts have been developed and evaluated to provide explicit communication to VRUs from the vehicle. Compared to internal HMIs, these external interfaces can communicate information directly to VRUs. However, there is currently no consensus regarding the optimal mode of communication to achieve the desired effectiveness of an eHMI [4,35,36].

### 3.4. Visual eHMIs

Visual eHMIs use visual cues to communicate with other road users; they may use anthropometric (human-like) elements, explicit text, recognizable traffic symbols (i.e., stop signs), or more abstract signals to achieve communication. However, there are also limitations to the accessibility and feasibility of visual eHMIs; they will not provide sufficient information to the visually impaired who are a particularly vulnerable group in traffic [4].

#### 3.4.1. Text-Based eHMIs

Using a video-based study, Ref. [65] found that text-based eHMIs appear to be more easily understood than more abstract displays. However, text-based eHMIs require more visual attention and translation if used in multiple countries, which can be costly and time-consuming [66,67]. In an online survey, Ref. [66] evaluated how different eHMIs influenced pedestrians’ willingness to cross the road in front of an AV. The difference between text and an abstract light-bar, different eHMI colors, different message perspectives, and positions of the eHMI was evaluated. Text-based eHMIs were found to be more effective than light-bar eHMIs regardless of the color, position, or message perspective of the eHMI. However, there are limitations as the eHMIs were not tested in real traffic where there are high visual demands [68], indicating that the results may not have reflected real-world behavior.

#### 3.4.2. Icon-Based eHMIs

Unlike text-based eHMIs, icon-based traffic signals can overcome natural language barriers, transcend cultural borders, and be more conspicuous and legible from a greater distance [66,67]. However, in a picture-based online study, Ref. [69] has argued that first-time interpretation of icons may take longer than textual information as the effectiveness of icons depends on prior exposure. Furthermore, the concreteness of an icon partially determines its effectiveness; more abstract icons may be non-intuitive and may lead to information overload if road users need to decipher their meaning [68]. Reference [70] utilized a “green man/yellow hand” (GMYH) concept to signal that it was safe to cross (green man) and for the pedestrian to wait (yellow hand) in a virtual reality study. This concept worked independently of red/green color blindness, language, and reading skills. The GMYH concept is often used in pedestrian traffic lights, and the familiarity of this concept may allow participants to decipher the meaning of this eHMI quickly. The results exhibited that the GMYH concept is preferred over the two anthropomorphic interfaces evaluated.

#### 3.4.3. Anthropomorphic eHMIs

Anthropomorphic gestures employ elements of human appearance to communicate pedestrian recognition or vehicle intention to other road users. This may include facial expressions (smile or frown), eyes (direct gaze or closed eyes), or gestures such as waving. While anthropometric features have been found to promote likability and trust, it is debated whether they lead to safer and more efficient communication than non-anthropometric gestures [68]. Reference [67] had promising results when utilizing a Virtual Human Character (VHC) on the windshield of the car. These researchers evaluated how well a VHCs facial expression and gaze direction could support appropriate crossing decisions.

Using Virtual Reality, Ref. [70] compared two different anthropomorphic interfaces to an icon-based interface (GMYH). The two anthropomorphic interfaces evaluated were a humanoid ‘robot’ display that waved at pedestrians and a smile/neutral face concept that has been used in industry. A smile and a wave indicated that it was safe to cross, while a neutral face and no wave signaled the pedestrian to wait. While crossing decision time decreased regardless of the design, the ‘robot’ had the least significant effect. This is in line with findings from [64,70,71,72] which demonstrate that current research into anthropomorphic concepts is discouraging. However, Refs. [67,73] have had more promising results with interfaces that were successful at expressing the vehicle’s intention and supporting the appropriate action from participants.

#### 3.4.4. Abstract eHMIs

Abstract messages, most commonly communicated via light patterns, have been one of the most popular approaches in eHMI research, likely due to the ease of implementation. However, these messages can be unintuitive and require explanation or training [67]. In a VR study, Ref. [74] investigated how quickly a novel eHMI concept (Slow Pulsing Light Band–SPLB) effectively communicated yielding intention to a pedestrian when compared to a well-recognized message (flashing headlights) and a baseline condition (no eHMI). Compared to the SPLB and the baseline condition, the flashing headlight had higher visibility and led to earlier crossing decisions. Furthermore, participants were exposed to the SPLB for a block of trials before the eHMI had an effect on the crossing decisions when compared to the baseline. These results indicate that the familiarity of the flashing headlights required little learning and effected crossing decisions upon first viewing, while novel abstract eHMIs will require a learning period before they can reach full efficacy.

#### 3.4.5. eHMI Location

Additionally, the placement of existing visual eHMIs is diverse. Locations include on the vehicle (i.e., windshield, roof, grille, and the body of the vehicle), projection onto the road, or in the environmental infrastructure (i.e., traffic lights and smart sidewalks). Projected eHMIs have the ability to display a varying range of visual messages on a display surface limited only by the projector used. However, projections are difficult to see in daylight conditions, on rough surfaces, or in the presence of multiple vehicle projections [75]. In an online survey evaluating several different eHMI concepts, Ref. [68] found that a projection of a zebra crossing was found to be the clearest among non-textual eHMIs. This may be due to this representation being common worldwide. However, as the eHMIs evaluated were taken from the industry, some were presented without traffic context. Ref. [69] uncovered that traffic context has an impact on the comprehensibility of eHMIs; therefore, when utilizing surveys to evaluate the clarity of eHMIs, care must be taken to provide relevant context. In an online study, Ref. [66] evaluated how different eHMIs influenced pedestrians’ willingness to cross the road in front of an AV. They evaluated the difference between the eHMI designs, colors, message perspectives, and positions of the eHMI. Interfaces on the grille and the windshield were found to be more effective compared to the windshield, possibly because the angle of the windshield makes the eHMI less visible in certain situations.

#### 3.4.6. Color of Displays

There are legal constraints when it comes to the color of visual eHMIs, as outlined in the SAE J578 Standard and the UNECE R-65 Regulation [76,77]. For example, the UNECE forbids red lights at the front of the vehicle [78]. Recently, cyan has been explored in several eHMI concepts due to its high visibility and its current lack of specific meaning in traffic [66]. However, white, red, green, yellow, and purple have also been explored. In an online study, Ref. [66] evaluated how different eHMIs influenced pedestrians’ willingness to cross the road in front of an AV. The differences in perceived safety, when presented with differing eHMI designs, were evaluated. Participants indicated that they felt equally safe when presented with green and cyan interfaces, and were less willing to cross when presented with a red interface. This indicates that cyan may be interpreted similarly to green, and that red has the potential to signal that a pedestrian should not cross the road. However, Ref. [68] in an online survey uncovered that the content of a message was more persuasive than the color and that the color of the eHMI acted as a ‘reinforcer’ of the message if the text and color were congruent.

### 3.5. Auditory eHMIs

Auditory eHMIs utilize sounds to achieve communication. This modality has been under-studied within the eHMI literature when compared to visual eHMIs. Messages may be communicated through speech or abstract non-speech sounds.

#### 3.5.1. Speech-Based eHMIs

Similarly to text-based visual displays, eHMIs using speech are easily understood due to people’s ability to associate the words spoken with the information being conveyed. However, spoken word eHMIs will require translation when deployed in multiple countries. Furthermore, spoken words will generally take longer to process as the meaning can only be fully understood once all the words have been spoken [79].

#### 3.5.2. Abstract Sounds

Abstract sound signals can come in several different forms, including auditory icons an abstract earcons. Auditory icons are sounds developed to represent a commonly recognized sound related to the activity (i.e., a car horn represents a car). Auditory earcons take advantage of people’s prior knowledge to make connections between sounds and their intended messages [79]. Conversely, abstract earcons are synthesized sounds that have no direct relationship to the information [79]. On the other hand, these abstract sounds may be non-intuitive and lead to information overload if the road users cannot initially understand their meaning. Additionally, auditory-based eHMIs may be covered up by ambient noise. Similarly to visual eHMIs, auditory eHMIs have an accessibility problem as they will not provide information to the hearing impaired; however, they will still receive implicit communication cues from the vehicle movement.

### 3.6. eHMIs on Personal Devices

eHMIs delivered via personal devices have been proposed for communication through wearables, phones, tablets, and on bicycles. Compared to visual and auditory eHMIs, this type of eHMI is not widely researched in the AV-VRU literature. Reference [80] noted that sending haptic-based signals through a mobile phone may increase the safety of pedestrians by alerting them to potential hazards when they are distracted by their mobile phones. However, the implementation of these signals onto personal devices may cause privacy concerns. Additionally, requiring VRUs to purchase a wearable, or other new devices, to communicate with the AV may result in issues of accessibility due to the increased costs of purchasing new technology.

Using Virtual Reality, Ref. [80] examined the effect of text message alerts on crossing behavior in pedestrians distracted by their phones in conventional traffic. While the participants receiving the alerts selected larger gaps in traffic to cross the road, they also looked at the road less than both the control (non-distracted) group and the group without alerts. This indicates that the group with alerts relied on the messages to make their decisions, which may reduce their situational awareness, and thus their ability to respond to failures or unexpected events. Wearables have also been utilized in other use cases to communicate information to vulnerable road users. Of note, haptic feedback has been utilized to assist in navigation. Reference [81] assessed the effectiveness and acceptability of using haptic instructions communicated via wristband to assist older pedestrians in navigating an unfamiliar city. The results indicated that vibrotactile feedback reduced reaction time and allowed pedestrians to focus on elements in the environment that were important to the task. However, these messages may require a learning phase and can only be effective when the feedback is perceived and understood. The research from this use case may be transferrable to the field of AV-VRU interaction.

### 3.7. Message Perspective

**Figure 5:**
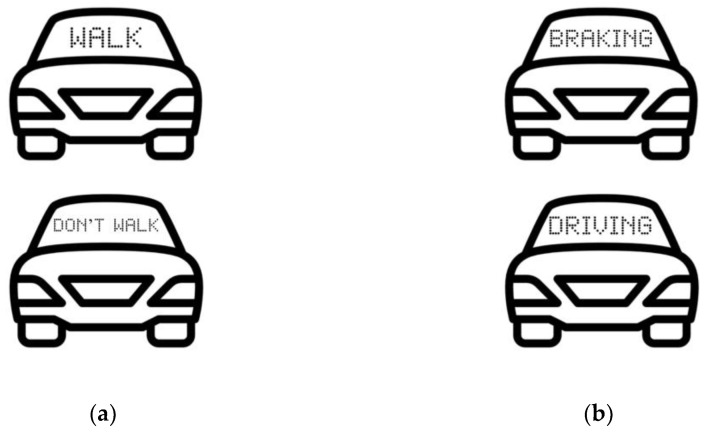
Potential eHMIs from different message perspectives: (**a**) Egocentric eHMIs (WALK/DON’T WALK); (**b**) Allocentric eHMIs (BRAKING/DRIVING).

An additional challenge in designing eHMIs is identifying the type of information that is necessary to communicate to VRUs and which perspective to communicate it from. According to [65], an egocentric message is a directive that addresses the road user so they can interpret the message from their own perspective. Egocentric messages communicate a call to action by directing road users on what to do (Figure 5a). Conversely, an allocentric message is a message that the road user has to interpret from the Avs’ perspective [65]. Allocentric messages communicate the intention or action of the AV rather than instructing the road user on what they should do (Figure 5b).

Research suggests that participants prefer direct information about what they should do (egocentric message) over information about the vehicle’s status or intention (allocentric message) and were more inclined to make appropriate crossing decisions when that information was presented [65,68]. Egocentric messages generally have low ambiguity which results in high clarity scores, while allocentric messages are open to multiple interpretations [65]. Furthermore, this may be due to egocentric bias, where people are more naturally able to interpret messages that pertain to themselves, and adjusting to a different perspective requires time and effort [65,68]. While haptic, anthropomorphic, and abstract may not be effected by the message perspective, it is important to note that road users may interpret them egocentrically with the absence of a specific perspective. This is further demonstrated when participants in a video-based study [65] were shown ambiguous messages (STOP, GO), and they interpreted them egocentrically. However, directing VRUs to take action could cause liability issues if the VRU complies with the eHMI instructions and becomes involved in an accident as a result. To further prove this point, Ref. [56] utilized virtual reality to evaluate whether eHMIs made pedestrians careless in traffic. Using three different text-based eHMIs, the researchers determined that the eHMIs that communicated the vehicle’s yielding intention (‘I will stop’) and gave the participants a directive (‘After You’) made the participants careless as they felt safe to cross without checking the surrounding traffic. However, while virtual reality creates a strong sense of presence and immersion, participants are likely more inclined to exhibit risk behavior when there is no real risk of physical injury [70]. Although these eHMIs made pedestrians careless, they still indicated a high degree of perceived safety. In the UK, the Highway Code forbids drivers to signal pedestrians and direct them to cross as it could be dangerous if another vehicle approaches [82]. If allocentric messaging is used to mitigate this effect, more research needs to be conducted to identify which driving states are essential to be communicated.

### 3.8. Intended Target

Most eHMI concepts evaluated in the literature have aimed to communicate with one pedestrian. Of the papers reviewed, only [79,83] proposed eHMI concepts to communicate with cyclists even though cyclists may exhibit different behaviors and perceptions than pedestrians. As such, the ideal communication strategy may differ between the two road user groups and needs to be further explored.

### 3.9. Accessibility

In a review of publications in the field of external communication of AVs, Ref. [84] uncovered that based on six principles of universal design, the needs of people with impairments were largely unevaluated and thus unmet. The principles included providing a means of use and making the design appealing for all users, accommodating varying levels of literacy and language skills, using multiple modes to present similar information, providing compatibility with a variety of devices, and providing a clear line of sight for people that are sitting and standing. It is necessary to take these principles into account from the start when designing eHMIs.

### 3.10. Evaluation of eHMIs

There are multiple ways to evaluate different eHMI concepts. However, it if difficult to compare individual designs, as different methodologies and metrics are used to measure their efficacy. Furthermore, several eHMI concepts have not been evaluated because they are limited to demonstrations for promotion purposed or proof-of-concept prototypes [4].

#### 3.10.1. Methodology

Virtual reality (VR) is an immersive technology where users experience a simulated environment through visual and auditory inputs. VR allows researchers to test fully functional prototypes within a simulated and interactive traffic environment without considerable risks to participants while still eliciting emotional and physical reactions similar to those in the real world [48,85]. Popular simulation platforms include computer-based setups, Cave Automatic Virtual Environment (CAVE) simulators, and VR head-mounted displays (HMD) (such as the Oculus Rift). However, there are various side effects associated with the use of VR, including simulator sickness which can cause dizziness, discomfort, nausea, and headache [86].

Field studies, often completed using Wizard-of-Oz techniques, is a method that is commonly used to get more natural and spontaneous behavior of participants. In a proof-of-concept experiment, Ref. [28] concluded that this method reliably creates the appearance of a driverless vehicle. While these studies have a high degree of realism and validity, they are costly and time-consuming. There are also limitations to their replication and adoption due to the difficulty of getting ethical approval and the lack of control over experimental variables [87]. Additionally, as this methodology often takes place outside of a laboratory setting, it may limit what behavioral data can be collected, particularly if naturalistic observation is utilized.

Questionnaire studies often utilize pictures or videos to introduce and compare different eHMI concepts, as well as gather measures of participant satisfaction, trust, willingness to cross, and eHMI clarity [27,66,68,69,88,89]. While these questionnaires are a beneficial first step in eHMI evaluation due to the wealth of data they can produce relatively inexpensively, they may not represent real-world behavior.

#### 3.10.2. Dependent Variables

While several studies employ similar metrics to evaluate eHMIs, the definition and the method of collecting seemingly similar variables may vary between studies [86]. Trust, perception of safety, mental workload, and crossing behaviors are overviewed below. Within the literature, trust has been identified as an important aspect of AV-VRU interaction; however, there is currently no consensus regarding the appropriate instruments to measure it [86]. Trust influences decision making and a person’s willingness to rely on automated systems. While it is easy to administer Likert scales to measure trust, they may have limited validity as they may not measure all aspects of trust. Standardized scales do aim to evaluate multiple dimensions, but as several standardized scales have been utilized across the literature, it limits the comparability of trust across results. One of the most commonly cited is the Trust in Automation scale [90]; however, it has been found to have a positive bias when presented in its original order [91]. Additionally, when measuring trust, it is necessary to ensure that the participant is not responding to the questionnaire thinking about a future version of AVs [87].

The perception of safety is also a frequently utilized dependent variable often measured using bespoke scale methods that include Likert scales and forced yes/no responses. Furthermore, through the use of a motion capture suit in a VR experiment, Ref. [92] used forward gait velocity as a measure of perception of safety. Studies on affective body language have shown that when presented with a negative stimulus, gait velocity will be reduced [92]. The perception of safety can also be measured by the participants’ willingness to cross in front of an AV. In a simulated indoor environment, Ref. [48] evaluated the willingness to cross as a timed duration of how long it took before pedestrians felt unsafe to cross by instructing participants to hold down a button for as long as they felt safe to cross. The willingness to cross can also be measured through bespoke scales. 

The mental workload can be measured through both subjective and objective metrics. The NASA-TLX subjective workload assessment tool is deemed reliable to measure mental workload. The NASA-TLX measures six dimensions on a 21-point scale, which are as follows: mental demand, physical demand, temporal demand, performance, effort, and frustration [93]. The objective mental workload can be evaluated through the use of eye tracking. In a video-based study that utilized eye tracking, Ref. [65] extracted pupil dilation and the number of saccades to indicate task difficulty and how many eye movements the participants made to reach a decision, respectively. Similarly, Ref. [94] extracted mean fixation duration and saccades to evaluate mental workload. 

Crossing behavior can be evaluated through a variety of metrics, including crossing initiation time, time to cross, and gap acceptance [7,56,62,70,74,75,80,87,94,95,96]. These are often calculated using video analysis or motion capture data. Gap acceptance, or the critical gap, is also a metric used to evaluate the crossing behavior. Gap acceptance can be defined as the gap in traffic that the participant is willing to accept between themselves and the vehicle to ensure crossing.

#### 3.10.3. Independent Variables

The independent variables utilized throughout the literature can be broken down into four categories as follows: environmental factors, vehicle factors, eHMI factors, and participant factors. Environmental factors have been utilized to understand how road environments or differing times of day influence the effectiveness of an eHMI. Vehicle factors include yielding behavior, vehicle size, and vehicle type. eHMI factors include the presence of an eHMI, the design of an eHMI (color, modality, location, etc.), and the communication strategy of the eHMI. Lastly, participant factors have been utilized to evaluate how different genders, age groups, and cultures have affected eHMI effectiveness.

### 3.11. VRU–Vehicle Communication in Shared Spaces

In a questionnaire conducted in three different locations where automated vehicles were deployed, Ref. [36] reported that pedestrians had a lower perception of safety when interacting with an automated vehicle in environments without lane markings. The speed of the vehicle was not a concern, likely due to the low-speeds that characterize shared spaces [36,37]; however, there was an overall agreement that an automated vehicle should have some form of external communication regarding the vehicle’s other actions. However, in line with the research on conventional streets, there is no clear agreement on the ideal modality to supply this information. Similarly, Ref. [97] conducted an online survey to uncover what information VRUs utilize to make decisions in their interactions with vehicles in shared spaces. Overall, pedestrians indicated that hand gestures, head nods, and eye contact with the driver were the most important, while cyclists indicated that the most important form of communication was turn signals and car movement.

While participants in [36] agreed that external communication was necessary, Ref. [37] found that external communication was rarely used in pedestrian-conventional vehicle interactions in an observation of a shared space (a car park). However, when compared to a conventional road, external communication is more prevalent in a shared space; external communication occurred in 17% of the recorded pedestrian-driver interactions within the shared space in [37], compared to the 4% recorded on a conventional road in [98]. The elevated levels of external communication may be due to the ambiguity of the interactions due to the lack of formal traffic rules within a car park. Furthermore, Ref. [9] reported that when priority was unclear, pedestrians commonly utilized explicit communication. Additionally, as found in [29,36], the lack of formal traffic infrastructure may contribute to the ambiguity of the interactions. In an interview with cyclists in the Netherlands and Norway, it was noted that cycling within a busy traffic environment requires a high mental workload resulting in more difficultly predicting other road users’ intentions [52]. In order to overcome this uncertainty, cyclists indicated that they use a mix of explicit communication, including eye contact and hand gestures, and implicit motion cues. However, which communication method—implicit or explicit—was relied on more differed between participants. Conversely, pedestrians in a shared area comfortably used the shared areas and were often engaged in their mobile phones or conversations [99]. While these actions support the objective of shared spaces, they lead to pedestrians becoming less aware of their surroundings and thus engaging in risky behaviors. As such, any communication method implemented into shared spaces needs to be carefully designed to support safer behavior while not undermining the shared space objectives [99].

### 3.12. eHMIs in Shared Spaces

As outlined in the previous section, interactions, and communication between road users often differ between shared spaces and conventional roads. The differences in behavior between shared spaces and conventional roads in [48] highlight the necessity of taking infrastructure into account when investigating the interactions and communication between AVs and VRUs. These differences raise the question of whether eHMIs will need to communicate differently in shared spaces to resolve ambiguities that may arise. In spite of this, there currently exists little research evaluating eHMIs within shared space environments.

#### 3.12.1. eHMI Technologies

Of the papers reviewed, 11 utilized abstract visual eHMIs, with a few utilizing anthropomorphic or icon-based eHMIs. Based on survey data examining what information road users wanted vehicles to communicate Ref. [97] proposed a unique multi-modal (visual and auditory) concept that has not yet been evaluated. Lastly, Ref. [100] utilized three different AR-based eHMIs that provided information about the moving behavior of vehicles (traffic augmentation), the gap between the cyclist and the vehicle (smart bicycle path), and a warning signaling a potentially dangerous situation (warning sign). Results showed that the smart bicycle path and traffic augmentation eHMIs increased information quantity and quality when compared to the warning sign and baseline condition. These results indicate that AR may be a promising approach to facilitate AV-VRU communication. While AR is a commonly suggested and promising solution for AVs to communicate with cyclists [100], several interviewees in [52] aptly noted that requiring an on-bike eHMI in order to increase their safety will make bikes less accessible to the majority due to the increased cost of purchasing additional technology. Furthermore, a majority of studies focused on AV-pedestrian interactions, with only [52,100] as the exception focusing on cyclists.

#### 3.12.2. Methodology

While the majority of the reviewed papers utilized video-based surveys to evaluate their eHMI concepts, a few [101,102,103,104] utilized VR or field-based testing. As previously discussed, while questionnaires are a good first step in the evaluation, they often do not reflect real-world behavior due to the lack of ecological validity. However, in a manipulation check after a Wizard-of-Oz interaction, very few participants were convinced that the vehicle they interacted with was driving automatically. Interestingly, Refs. [52,97] utilized a survey and interviews to obtain road users’ input on the design of eHMIs. In interviews with cyclists, participants expressed skepticism regarding whether they would be comfortable interacting with automated vehicles in shared environments, expressing concern over whether an AV would understand informal traffic rules [52]. As a result, one of the most reoccurring topics in the interviews was the need for acknowledgment from the AV that they had been seen.

#### 3.12.3. Findings

Findings regarding the clarity of abstract eHMIs in shared spaces were mixed. In online video-based surveys, Refs. [101,102,103,104] used similar light-based eHMIs to investigate pedestrians’ interactions with differently sized AVs. The eHMIs indicated differing combinations of the vehicle’s automation status (VAS), the vehicle’s intention, and the vehicle’s perception (VAS + intention, VAS + perception, and VAS + intention + perception). Participants perceived all the communication strategies as clear even when presented for the first time [101,102,103,104]. This is in direct opposition to the findings in [105] where participants thought light-based signals were unintuitive in a Wizard-of-Oz study. Furthermore, Ref. [106] reported that a pedestrian’s mental model does not evolve after an initial interaction with an automated vehicle. As such, care must be taken to properly design the eHMIs to avoid the creation of an inappropriate mental model.

Results from several studies indicate that it is necessary to ensure that vehicle kinematics are well-coordinated to facilitate appropriate levels of trust and feelings of safety. In [101,103], when dynamic eHMIs indicated yielding intent which was contradictory to the vehicle kinematics, participants still reported increased perceived safety indicating that participants were over-reliant on the eHMI. Conversely, in [107], after experiencing an eHMI failure condition, participants’ trust in the eHMI and their perception of safety declined significantly. The results in [108] indicated that implicit cues are equally important as explicit information in facilitating clear communication because when pedestrians are presented with multiple messages, they may filter for information that is relevant to their goals and ensure their safety.

Results from [48,98,101,102,104] demonstrated that pedestrians feel safer when interacting with small and slow-moving vehicles. This can be seen in a field study where pedestrians were observed crossing the road even when the message on the eHMI indicated to stop, likely due to the size and speed (<5 m/s) of the vehicle [109]. However, Refs. [102,104] demonstrated that vehicle size did not influence communication needs, indicating that communication strategies can be transferable between vehicles of different sizes.

Several studies have evaluated the effect of eHMIs on participants trust levels. Using a naturalistic Wizard-of-Oz approach, Ref. [105] evaluated how different light-based signals communicated information about the vehicle’s state and future maneuvers, and advice-based information on a University campus. Results revealed that the light signals were not intuitive and that participants found the eHMIs only partially trustworthy, resulting in a decreased perception of safety when compared to interacting with a manual vehicle. Similarly, Ref. [109] evaluated how a visual eHMI (anthropomorphic and abstract) affected pedestrian behavior in a shared space on a college campus. Video analysis was used to identify pedestrian behavioral patterns. Results indicated that neither eHMI had a statistically significant effect on pedestrian crossing behavior. Furthermore, Ref. [110] indicated that if one eHMI was superior in communicating the vehicle’s intentions, the participant would be willing to step into the shared space for a longer duration. The results of the study indicated that an ‘absolute’ concept was superior in maximizing the time duration where the participant felt comfortable stepping into the space. However, the levels of trust and acceptance were not significantly different between the two eHMIs. Moreover, the summed total trust score measured using [90] indicated that neither eHMI was particularly trustworthy. Additionally, in scenarios where participants were unsure of the vehicle’s intentions, they tended to rely on more implicit cues.

Findings regarding the clarity of abstract eHMIs were mixed. In online video-based surveys, Refs. [101,102,103,104] used similar light-based eHMIs to investigate pedestrians’ interactions with differently sized AVs. The eHMIs indicated differing combinations of the vehicle’s automation status (VAS), the vehicle’s intention, and the vehicle’s perception (VAS + intention, VAS + perception, and VAS + intention + perception). Participants perceived all the communication strategies as clear even when presented for the first time [101,102,103,104]. This is in direct opposition to the findings in [105] where participants thought light-based signals were unintuitive in a Wizard-of-Oz study. However, very few participants were actually convinced that the vehicle was driving automatically. 

More detailed findings of the papers reviewed can be found in Table 1.

## 4. Discussion

The eHMIs are a key design solution to improving AV-VRU interactions that have been explored throughout academia and industry. In order to answer the research questions on the knowledge and the gaps in the literature, we reviewed AV-VRU communications, the general findings of eHMI use on conventional roads, and the findings of eHMIs on shared roads. The three main insights of that literature review are presented and discussed in the following paragraphs.

While we conclude that eHMIs will most likely assist in improving AV-VRU interactions, they will need to be well-designed and evaluated to be effective. However, there is currently a gap in the research investigating the negative effects of eHMIs. In a review, Ref. [8] summarized the potential drawbacks of eHMIs which included competing with other visual cues in the environment, the intuitiveness of the display, and the overreliance on the technology. While it is apparent that there are negatives to eHMIs, many studies do not report on the drawbacks that the proposed eHMIs could introduce.

Most eHMI concepts that have been evaluated aim to communicate with pedestrians, with little research focusing on other vulnerable road users, such as cyclists, despite cyclists often exhibiting different behaviors than pedestrians. As such, understanding if and how eHMIs should differ between pedestrians and cyclists and other VRUs would ensure that all road users get the expected safety benefits of automated vehicles. Furthermore, there is no consensus on what information is essential to communicate to VRUs, what modality (icon, text, light-based, auditory, etc.) is most effective, or the ideal communication strategy. We conclude that identifying the best practice for eHMI to develop design recommendations for vehicle manufacturers is a major gap; having different designs on every automated vehicle may confuse VRUs, as the same icon, or abstract signal, may have different meanings depending on the manufacturer and could be misinterpreted by road users [113]. This would require the VRU to identify the vehicle manufacturer before understanding the meaning of the eHMI. 

While the papers reviewed have evaluated eHMIs in shared space environments, few address the truly complex and ambiguous nature of shared spaces. Similarly to evaluations of eHMIs on conventional roads, a majority of eHMIs in shared spaces have been evaluated in the case of one vehicle and one pedestrian, with limited other traffic [114]. In reality, shared spaces will often have several road users interacting in a dynamic fashion. Furthermore, from the papers selected, none of the researchers specified the type of shared space. Creating an agreed-upon taxonomy of shared spaces would be beneficial to allow researchers to accurately describe the environments they are researching within. Secondly, a majority of the papers reviewed utilized abstract light-based eHMIs with conflicting results. Moreover, several studies reviewed utilized surveys to evaluate the efficacy of their eHMI concepts. As previously discussed, while this is a beneficial first step, the lack of ecological validity limits these results. Further research should be done utilizing VR or field studies to confirm the results of these studies. Lastly, based on the literature available it is difficult to confirm whether communication needs to differ between conventional roads and shared spaces. While research has confirmed that behavior differs between the environments, the differing evaluation variables and the lack of shared space research make it difficult to compare findings.

## 5. Conclusions and Future Research

Before automated vehicles can be successfully integrated into future traffic, more research needs to be done into establishing an effective eHMI that will be able to communicate to diverse road users and in complex situations. As previously stated, eHMIs have been primarily evaluated in simple interactions and environments despite the reality being much more complex. In particular, shared spaces are dynamic environments where several road users may be interacting at once, resulting in it being unclear who has priority. Future evaluations should aim to create more complex scenarios in order to ensure that eHMIs will be effective in practice in all road environments. Additionally, this review has highlighted the lack of research into non-visual eHMIs, particularly in the context of shared spaces. Future research should explore haptic and auditory solutions, as well as multi-modal solutions, in order to not alienate visually impaired VRUs.

This work has also revealed the existence of several challenges which provide a direction for future research from a technical perspective. One challenge that has not been addressed is the technical feasibility of implementing eHMIs into the vehicle design. Currently, most eHMIs are implemented virtually or added as an addition to the already existing vehicles. The literature has emphasized the importance of the eHMI and the vehicle kinematics being well-coordinated. As such, this highlights the need for the eHMI to be effectively integrated into the vehicle design in order to suitably coordinate the behavior of the eHMI to the implicit cues of the vehicle. Furthermore, a majority of eHMIs have been evaluated in daylight and good weather conditions. However, the effectiveness of an eHMI can be hindered by environmental conditions (i.e., projections are difficult to see on rough surfaces or in crowded environments, audio is difficult to hear over ambient noise, etc.). This introduces a second technical challenge of ensuring the eHMI is effective in all conditions. The scope of the research will need to expand to overcome the technical challenges of eHMI implementation. Finally, future research should shift towards experimental designs with more consistency in the measures collected, in the eHMI design guidelines used, and in the ecological validity of the use cases investigated (i.e., reflect reality rather than the lab environment). This review has underlined that more research needs to be completed to determine if the communication needs of vulnerable road users truly differ between shared spaces and conventional roads when interacting with automated vehicles.

## Figures and Tables

**Table 1:** General findings of the papers reviewed researching eHMIs for shared spaces.

Ref	Research Question	eHMI Description	Methodology	General Findings
[52]	Interview topics included Current traffic interactions and experiences; The future of cycling when there is no longer a human driver; The perfect future bicycle.	N/A	Interviews (Semi-Structured)	Participants expressed skepticism regarding whether they would be comfortable interacting with automated vehicles in shared environments, expressing concern over whether an AV would understand informal traffic rules. One of the most reoccurring topics in the interviews was the need for acknowledgment from the AV that they had been seen. Generally agreed that the AV should signal both intent and perception, preferences for the design varied, ranging from a light strip to an auditory interface. The most common design strategy that was suggested was an on-bike device. One interviewee stated that receiving more information about the traffic environment “helps with uncertainty of the driving environment.”
[97]	What information and communication is expected by VRUs Do VRUs expect traditional communication methods Do different road users need different communication methods	N/A	Video-Based Survey	Pedestrians utilized turn signals, brake lights, and car movement to gain information. Cyclists preferred turn signals, car movement, eye contact, and the car horn. Compared to pedestrians, cyclists expect communication from manual vehicles in more straightforward ways. Proposed a multi-modal eHMI concept that communicated awareness of the situation, perceptions of risks, decision making, and positive feedback.
[110]	Evaluated how eHMI designs influenced pedestrians’ willingness to step into the shared space	Absolute: Icon, Blue, Projection, Allocentric (projection of arrows of vehicle’s intended path) Relative: Abstract, Green/Amber/Red, Vehicle Body, Allocentric (light-based signals similar to conventional turn signals) Targeted Pedestrians	Simulated Environment IVs: eHMI Strategy, Vehicle Route DVs: Perceived Safety, Trust	Indicates that the absolute concept was superior in maximizing the time duration where the participant felt comfortable stepping into the space. Levels of trust and acceptance were not significantly different between the two eHMIs. Measured using [90], the summed total trust score indicated that neither eHMI was particularly trustworthy. In scenarios where participants were unsure of the vehicle’s intentions, they tended to rely on more implicit cues.
[101]	Are results transferrable between differently sized vehicles What are the negative effect of mismatched signals	‘Static’ *eHMI:* Abstract, Cyan, Vehicle Body, Allocentric (automation status) ‘Dynamic’ *eHMI:* Abstract, Cyan, Vehicle Body, Allocentric) Targets Pedestrians	Online Survey (Video-Based) IVs: eHMI Status, Vehicle Behavior DVs: Willingness to Cross	Participants were only willing to cross when the information about the vehicle’s behavior matched the eHMI’s message. Participants relied on the information of the dynamic eHMI more than the vehicle’s behavior.
[102]	Investigate the effect of vehicle size Investigate information richness level Investigate 3 different dynamic eHMIs	Static eHMI: Abstract, Cyan, Vehicle Body, Allocentric (automation status (VAS)) Dynamic *eHMIs:* Abstract, Cyan, Vehicle Body, Allocentric Targets Pedestrians	Online Survey (Video-Based) IVs: Vehicle Size, eHMI Strategy DVs: Perceived Safety, Self-Assessment Manikin (SAM), Usability	Dynamic eHMIs had a positive effect on perceived safety and affective evaluation. Indicated a high information richness level increased perceived safety, affective evaluation, perceived information quality, and usability. Dynamic eHMIs were clearer than the static eHMI indicating a higher information richness level is preferred.
[103]	Investigate the interplay between the eHMI message and the vehicle kinematics	Static’ eHMI: Abstract, Cyan, Vehicle Body, Allocentric (automation status) ‘Dynamic’ eHMI: Abstract, Cyan, Vehicle Body, Allocentric Targets Pedestrians	Online Survey (Video-Based) IVs: Vehicle Size, Vehicle Kinematics, eHMI Status DVs: Willingness to Cross, Trust, Perceived Safety	Highlighted the need for vehicle kinematics and the eHMI to be well-coordinated. Negative effects were found when the eHMI and vehicle kinematics were not well-coordinated. Participants were over-reliant on the eHMI—increased safety was reported even when yielding intent was contradictory. Participants had a higher willingness to cross when there was a dynamic eHMI compared to the static eHMI or no eHMI. Participants had a lower willingness to cross in front of a larger AV.
[104]	Investigate different eHMI communication strategies between two different vehicle sizes	Intention Based: Abstract, Cyan, Vehicle Body, Allocentric (yielding intention) Perception Based: Abstract, Cyan, Vehicle Body, Allocentric Combined: Abstract, Cyan, Vehicle Body, Allocentric Targets Pedestrians	Online Survey (Video-Based) IVs: Vehicle Size, eHMI Strategy DVs: Quality of Information, Perceived Safety, SAM	Participants had a lower feeling of safety in front of an automated bus Results indicated that the eHMI communication strategy is applicable across vehicle sizes. No significant difference between the three different eHMIs.
[111]	How an eHMI should be designed for differently sized vehicles Are the eHMI designs suitable for different age groups	‘Static’ eHMI: Abstract, Cyan, Vehicle Body, Allocentric (automation status) ‘Dynamic’ eHMI: Abstract, Cyan, Vehicle Body, Allocentric (VAS. VAS + intention, VAS + perception, VAS + intention + perception) Targets Pedestrians	Video-Based Survey IVs: Age group, information richness, eHMI design DV: Perceived Information Quality, Perceived Safety, SAM	eHMI designs with higher information richness (‘dynamic’ eHMIs) were preferred over no eHMI and static eHMI for all age groups. Young people (aged 16–24) felt safer and had higher information quality scores than elderly people (aged 65–74). Vehicle size has a significant effect on perceived safety and affective valence indicating eHMIs may be especially important with large vehicles.
[105]	Evaluated how different light-based signals communicated information How the development of perceived safety was influenced by participant’s age and mismatch between vehicle’s movements and eHMI signals.	Automation Mode: Abstract, Cyan, Roof, Allocentric Starting Mode: Abstract, Cyan, Roof, Allocentric Crossing Mode: Abstract, Cyan, Roof, Egocentric Targets Pedestrians	Field Study (Wizard of Oz) IVs: eHMI Strategy, Driving Condition DVs: Intuitiveness, Comprehensibility, Trust, Perceived Safety, Usefulness	Results revealed that the light signals were not intuitive and that participants found the eHMIs only partially trustworthy, resulting in a decreased perception of safety when compared to interacting with a manual vehicle. However, in a manipulation check conducted through interviews after the interaction, very few participants were actually convinced that the vehicle was driving automatically.
[112]	Investigated the effects of an eHMI malfunction (vehicle kinematics-eHMI mismatch) on participants’ assessment of the system. Investigated age-related effects	Automation Mode: Abstract, Cyan, Windscreen, Allocentric Crossing Mode: Abstract, Cyan, Windscreen, Allocentric Targets Pedestrians	Video-Based Survey IVs: Age group, eHMI Strategy, Vehicle Behavior DVs: Trust, Acceptance, Perceived Safety, Vigilance	Elderly participants indicated higher levels of trust, usefulness, and feelings of safety across all conditions than younger participants. After experiencing an eHMI failure condition, participants’ trust in the eHMI and their perception of safety declined significantly.
[107]	How do eHMI malfunctions affect the development of elderly participants’ assessment of trust and feeling of safety	Automation Mode: Abstract, Windscreen, Allocentric Crossing Mode: Abstract, Windscreen, Allocentric Targets Pedestrians	Video-Based Survey IVs: Vehicle Behavior, eHMI Strategy DVs: Trust, Perceived Safety	Participants reported increased trust from their initial rating after having experience with an eHMI. After experiencing an eHMI failure condition, participants’ trust in the eHMI and their perception of safety declined significantly.
[109]	Evaluated how a visual eHMI (anthropomorphic and abstract) affected pedestrian behavior in a shared space on a college campus.	Red-Green Sign: Abstract, Red/Green, Windscreen Open-Closed Eyes: Anthropomorphic, Windscreen Targets Pedestrians	Field Study (Video Analysis) IVs: eHMI Strategy DVs: Crossing Behavior	Results indicated that neither eHMI had a statistically significant effect on pedestrian crossing behavior. Furthermore, pedestrians were observed crossing the road even when the message on the eHMI indicated to stop, likely due to the size and speed (<5 m/s) of the vehicle. This is in line with the results in [48,101,102,103,104], indicating that smaller vehicle sizes and slower speeds result in greater feelings of safety.
[108]	Investigated the efficacy of eHMI communication in a complex urban mobility scenario	Abstract, Purple, Vehicle Body, Allocentric Targets Pedestrians	Virtual Reality (Head Mounted Display) IVs: Traffic Scenario DVs: Comprehensibility, Trust, General Experience	When pedestrians are presented with multiple messages, they may filter for information that is relevant to their goals and ensure their safety. Results indicated that implicit cues are equally important as explicit information in facilitating clear communication.
[100]	Evaluated 3 different AR-based displays	Traffic Augmentation: Augmented Reality (AR), Allocentric Smart Bicycle Path: AR, Red/Green, Egocentric Warning Sign AR, Allocentric Targets Cyclists	Virtual Reality (Head-Mounted Display) IVs: eHMI Strategy DVs: Positive/Negative Affect schedule (PANAS), Situation Awareness, Acceptance	Results showed that the smart bicycle path and traffic augmentation eHMIs increased information quantity and quality when compared to the warning sign and baseline condition. These results indicate that AR may be a promising approach to facilitate AV-VRU communication. While AR is a commonly suggested and promising solution for AVs to communicate with cyclists [100], several interviewees in [52] aptly noted that requiring an on-bike eHMI in order to increase their safety will make bikes less accessible to the majority due to the increased cost of purchasing additional technology.
[106]	Do pedestrians develop a mental model of automated driving systems after initial interaction? Is it dependent on the eHMI type?	High AT: Abstract, Cyan, Roof Low AT: Abstract, Cyan, Roof Targets Pedestrians	Field Test IVs: Automation Transparency (AT) (via eHMI) DVs: Mental Model	After three interactions with the automated vehicle, pedestrians’ mental model did not evolve in terms of functionalities, abilities, and system limitations after the initial interaction. Highly transparent eHMIs enhanced pedestrians’ mental model of the vehicle’s ability to communicate. Pedestrians’ misconceptions of the vehicle’s abilities may be induced by the eHMI.

## Data Availability

No new data was created or analyzed in this study. Data sharing is not applicable to this article.
